# Systemic inflammation is associated with increased risk of death in population with atherosclerotic cardiovascular disease and chronic kidney disease—a Danish national register study

**DOI:** 10.3389/fcvm.2026.1749835

**Published:** 2026-02-27

**Authors:** Jan Håkon Rudolfsen, Jelena Vukmirica, Pierre Johansen, Kasper Løwe Lundgren, Martin Bødtker Mortensen

**Affiliations:** 1 EY Parthenon P/S, Frederiksberg, Denmark; 2 Novo Nordisk Denmark A/S, Copenhagen, Denmark; 3 Department of Cardiology, Aarhus University Hospital, Aarhus, Denmark; 4 Department of Cardiology, Johns Hopkins, Baltimore, MD, United States

**Keywords:** atherosclerotic cardiovascular disease, cardiovascular disease, chronic kidney disease, C-reactive protein, inflammation

## Abstract

**Aims:**

Systemic inflammation (SI), indicated by elevated C-reactive protein (CRP) levels, is known to increase the risk of major adverse cardiovascular events (MACE) and mortality. This study aims to investigate the association between SI and mortality in the Danish population diagnosed with atherosclerotic cardiovascular disease ASCVD and chronic kidney disease CKD.

**Methods:**

We identified 19,159 individuals with incident ASCVD and CKD between 2012 and 2022 in Danish national health registers. SI was defined by at least two CRP measurements between 2 mg/L and 20 mg/L within a six-month period. Cox proportional hazards models were employed to assess the relationship between SI and mortality, adjusting for relevant confounders.

**Results:**

Among the cohort, 68% were observed with SI. SI were associated with significantly higher risk of mortality, with a hazard ratio (HR) of 2.06 (95% CI: 1.92–2.21) for death and 1.66 (95% CI: 1.57–1.77) for ‘MACE or death’. The results were consistent in all subgroup analyses and sensitivity analyses, including in men and women separately, and using different definitions of SI.

**Conclusion:**

This study demonstrates that SI is prevalent among patients with ASCVD and CKD being strongly associated with higher risk of mortality and MACE. These findings suggest that SI could serve as a valuable marker to identify patients with ASCVD and CKD who are at particularly high risk and may benefit from targeted preventive interventions.

## Introduction

Atherosclerotic cardiovascular disease (ASCVD) affects 500 million individuals worldwide and is the cause of about 19 million annual deaths (1). Meanwhile, chronic kidney disease (CKD) has a global prevalence of almost 700 million and is a risk factor for cardiovascular disease (2). In Western countries overall, 1%–2% of the population is diagnosed with both ASCVD and CKD and these individuals exhibits a particularly high risk of cardiovascular (CV) events or death (3). In Denmark, ASCVD and CKD are prevalent health conditions which affect 7% and 10% of the adult population, respectively (2, 4). In ASCVD, the process of atherosclerotic plaque formation is driven by local, chronic inflammation of the vessel wall (5, 6). Parallels can be drawn with CKD, where compromised renal function fosters a milieu conducive to systemic inflammation (SI).

While the combined impact of ASCVD and CKD on major adverse cardiovascular events (MACE) and death is well established (7, 8), a substantial residual risk remains even after addressing known cardiovascular risk factors such as hypertension, diabetes, obesity, and promoting lifestyle modifications including diet and avoiding tobacco use (9). This persistent risk may, in part, be driven by SI (10)—an all-encompassing inflammatory response extending beyond localised sites, and present in approximately 35% of adults in population-based studies (11).

The association between SI and risk of CV events is already established in population screening studies and in clinical trials (12–15). Recently, the updated European Society of Cardiology guidelines for chronic coronary syndrome list C-reactive protein (CRP) as a method for risk stratification for individuals with CVD (16).

While previous studies have investigated the impact of SI in individuals with ASCVD and CKD individually (17, 18), the association of SI with mortality and MACE in a real-world setting among patients with both ASCVD and CKD remains underexplored. Patients with both conditions are widely recognized as being at particularly high risk. The aim of the present study was to assess the association between SI and mortality, as well as SI and MACE, in this high-risk population, using Danish registry data. The findings contribute to a more complete understanding of the impact of systemic inflammation in this high-risk group.

## Materials and methods

Data from Danish national health and administrative registers were obtained from 1 January 1994 through 31 December 2022. All individuals in Denmark are registered with a unique 10-digit personal identification code, provided at birth or upon immigration. This identification code enables a secure and accurate connection across all data sources containing individual-level data. Since this study was not a clinical trial, did not include patient contact, and did not collect biological samples, under Danish legislation it did not require ethical review board approval (19).

### Identification of study population

Individuals diagnosed with ASCVD from 1994 onwards were identified by ICD-10 codes or procedural codes in the Danish National Patient Register (NPR) (22). Inclusion criteria are presented in Table 1. Within the population of individuals with ASCVD, CKD stages 3–4 was determined by eGFR in the range 15–59 mL/min/1.73 m^2^, as reported in the RLRR (23). Finally, individuals were required to be observed via at least two CRP tests fitting the criteria outline above, within six months, in the two years before or after index date.

**Table 1:** Definition of ASCVD.

Diagnosis or procedure	ICD-10 or procedure code
Myocardial infarction	ICD-10: I21
Coronary revascularisation	Procedure code: KFNG, KFNF, KFNA, KFNB, KFNC, KFND, KFNE, KFNH20
Peripheral arterial disease	ICD-10: I70–I47, I77
Ischemic stroke	ICD-10: I63, I64

Includes all sublevels of the specified ICD-10 codes and procedure codes according to the Nordic Classification of Surgical Procedures (NCSP).

The index date was defined as the date of diagnosis of ASCVD if the individual already had a diagnosis of CKD or the date of diagnosis of CKD if the individual already had a diagnosis of ASCVD.

### Exclusion criteria

The RLRR contains test results from 2011 onwards, with some regional variation in reporting. To ensure that only incident individuals were included in the study, all individuals diagnosed with CKD in the first year of reporting in the RLRR were excluded. Similarly, individuals diagnosed with ASCVD prior to, or in the first year of, reporting in the RLRR were excluded. Some hospital regions only started reporting after 2011. The first potential year of reporting for the individual was therefore decided by the region of residence, coinciding with when the region in question started reporting to the register. Consequently, the study population consisted exclusively of individuals newly diagnosed with both ASCVD and CKD from 2012 onwards.

Conditions of an inflammatory or a chronic nature are also thought to increase CRP levels. Hence, individuals with a diagnosis of HIV, hepatitis C, tuberculosis, inflammatory bowel disease or cancer, or with indications for transplant, were excluded. Criteria for identifying these conditions are provided in Supplementary Table S1.

### Identification of CRP test results

CRP test results were obtained from the Register of Laboratory Results for Research (RLRR). In Denmark, CRP is frequently included in routine blood tests performed in both primary care and hospital settings, meaning that many CRP measurements are obtained as part of general clinical evaluation. As CRP test results were obtained only when individuals sought healthcare services, and due to the mode of testing, however, some CRP test results had to be excluded.

It was not possible to distinguish high-sensitivity CRP tests from wide-spectrum CRP tests. However, modern laboratory equipment is capable of accurately identifying CRP levels <10 mg/L, despite using wide-spectrum CRP (wsCRP) assays (20). Two common CRP test methods (assays) used during the study period had a lower range of CRP = 2.9 mg/L and CRP = 4 mg/L. All tests corresponding to these values were therefore excluded, as they likely did not represent the true CRP level.

Furthermore, test results could have been observed in association with infections requiring antibiotic or antiviral medications. Hence, all tests observed seven days before or 30 days after collection of a prescription for antibiotic or antiviral medications were excluded. Data on the collection of prescriptions were obtained from the Register of Medicinal Product Statistics (21).

Moreover, ASCVD events can trigger an immune response which results in increased CRP. Hence, all test results observed in the 30 days following an ASCVD event were excluded.

### Defining systemic inflammation (SI)

SI has been defined as having one high-sensitivity CRP test in the range of 2 mg/L–20 mg/L (24, 25). This definition is applicable in screening studies or inclusion criteria for a randomised controlled trial. In this study, however, individuals are tested when seeking medical care. As there are multiple reasons CRP might be elevated when seeking medical care, an alternative criterion to define SI had to be applied.

Hence, to be as certain as possible that individuals included in this study actually have SI, SI was defined as individuals having at least two CRP tests in the 2 mg/L–20 mg/L range less than six months apart. The same requirement of having multiple CRP tests were applied for the group without SI as well, to mitigate the issue of potential misclassification due to transient elevations in CRP and to mitigate the potential issue of differences in testing frequency.

Furthermore, since the exact incidence date of SI cannot be determined, the six-month time period had to be observed within two years of the study index date.

### Study outcomes

Dates of death occurring between 1 January 2011 and 31 December 2022 were gathered from the Danish Register of Causes of Death (26). As a secondary outcome, the time from index date to either first ‘MACE or death’ was applied. MACE was defined by ICD-10 codes in the NPR as myocardial infarction (ICD-10: I21, I22, I23, including subgroups) or ischemic, haemorrhagic or undetermined stroke (ICD-10: H34.1, G45–46, I60–61, I63, I64) as primary diagnosis, or as all-cause mortality following the latter incidence of ASCVD or CKD (27, 28). As all individuals had established ASCVD at baseline, MACE events reflect subsequent (i.e., recurrent) cardiovascular outcomes.

Moreover, the results are put in context by an investigation of the presence of selected comorbidities at index date, and of the incidence of select comorbidities following index date. The select comorbidities were stroke, heart failure, type 2 diabetes, type 1 diabetes, chronic obstructive pulmonary disease, myocardial infarction and atrial fibrillation. The method of identification for each comorbidity is presented in Supplementary Table S4.

### Statistical analysis

The Cox proportional hazards model was used to quantify the hazards associated with SI following index date. The exposure of interest was SI (yes/no). The effect was estimated for the population as well as for subgroups. Hazard ratios for subgroups were estimated through the interaction of SI with the relevant subgroup indicator and by calculating the effect in the group using delta methods (29).

The Cox model was adjusted to avoid potential confounding in the SI/no-SI groups. The included covariates were separated into demographic, biomarker and comorbidity categories. A forward-stepping approach was applied to observe whether any of the categories had a major impact on the hazard related to SI. The final model was adjusted for age, sex, region of residence, education, history of heart failure, diabetes, triglyceride, HbA1c and cholesterol (LDL-c). Sensitivity testing, including additional adjusting for cardiovascular diseases (CVD) such as hypertension and atrial fibrillation, did not influence the inference from the model.

The Cox model validity was assessed by Schoenfeld Residuals. To ensure that the proportional hazard assumption was upheld, individuals were required to survive at least 10 days after index date to be included in the study.

The same approach was applied for the ‘MACE or death’ outcome. Cumulative probability plots for ‘MACE or death’, respectively, are presented alongside the hazard ratios.

Because the study contains up to a 10-year follow-up time, an individual's SI status may change from index date to the end of the follow-up period. To ensure that the results are not a consequence of SI classification timing, a model using repeated measurements of CRP is also presented. The model specification reflects the static model, but model residuals were clustered for each individual. In this model, the CRP values were stratified by categories <2 mg/L (reference), 2–<5 mg/L, 5–<10 mg/L and 10–<20 mg/L.

Finally, all analyses were conducted using two alternative definitions of SI: (1) a relaxed criterion, classified as one CRP test result of 2 mg/L–20 mg/L within two years of index date as SI, and (2) strict criteria, classified as three CRP test results of 2 mg/L–20 mg/L less than six months apart, within two years of index date, as SI.

## Results

**Figure 1:**
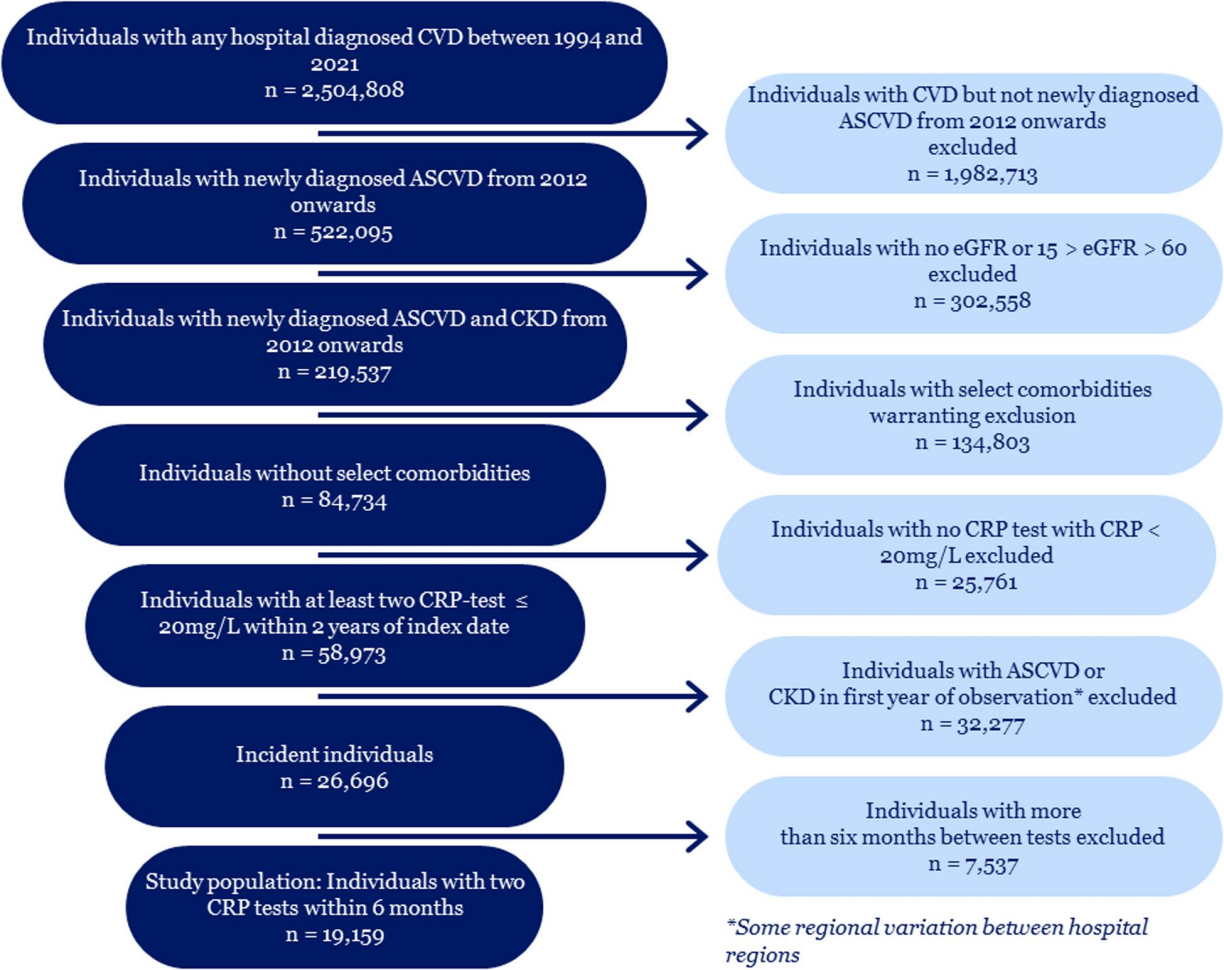
Flowchart of study population identification.

A total of 522,095 individuals were identified in the Danish registers as having ASCVD, in the period between 1994 and 2022. Among these individuals, 219,523 also had CKD stage 3–4 after 2011. Implementing the exclusion criteria related to comorbidities left 84,734 individuals with both ASCVD and CKD. After ensuring a sufficient number of CRP tests, and after excluding individuals from or before the first year of reporting in the RLRR, the final study population consisted of 19,159 individuals. Figure 1 illustrates the study population identification process.

### Prevalence of SI

Of the 19,159 patients, 13,036 (68%) had SI according to the definition of two valid measurements with values between 2 mg/L and 20 mg/L. In the no-SI population (*n* = 6,123, 32%), 30% of the CRP test results were above 2 mg/L.

The SI and no-SI populations did not have clinically significant differences with respect to age, sex, region of residence, education or cultural background (Table 2). Both groups included slightly more men than women, and most individuals were diagnosed with ASCVD prior to being diagnosed with CKD. The SI population had a higher burden of illness at index date, with more frequent history of hypertension, atrial fibrillation, COPD, heart failure and type 2 diabetes. This population did not have a significantly different history of stroke or acute myocardial infarction.

**Table 2:** Summary statistics at index date.

Characteristic	No SI *N* = 6,123	SI *N* = 13,036	*p*-value
Age	75,0 (11,02)	76,0 (11,30)	<0,001
Age group			<0,001
<60	630 (10%)	1.259 (9.7%)	
60–64	503 (8,2%)	927 (7.1%)	
65–69	710 (12%)	1.413 (11%)	
70–74	1.129 (18%)	2.133 (16%)	
75–79	1.148 (19%)	2.350 (18%)	
≥80	2.003 (33%)	4.955 (38%)	
Sex			0.034
Men	3.370 (55%)	6.961 (53%)	
Women	2.753 (45%)	6.075 (47%)	
First diagnosis			0.013
ASCVD	3.914 (64%)	8.090 (62%)	
CKD	2.209 (36%)	4.946 (38%)	
Region			<0.001
North Jutland	743 (12%)	1.605 (12%)	
Mid Jutland	1.228 (20%)	2.657 (20%)	
Southern Denmark	621 (10%)	1.816 (14%)	
Capital	2.660 (43%)	5.270 (40%)	
Zealand	871 (14%)	1.688 (13%)	
Education			<0.001
Bachelor's or equivalent	872 (14%)	1.538 (12%)	
Master's or higher	308 (5.0%)	492 (3.8%)	
Primary or unknown	223 (3.6%)	547 (4.2%)	
Secondary education	4.720 (77%)	10.459 (80%)	
Cultural background			0.7
Both Danish parents	5.730 (94%)	12.228 (94%)	
One Danish parent	384 (6.3%)	784 (6.0%)	
Non-Danish parents	9 (0.1%)	24 (0.2%)	
CRP test characteristics			
*N* test	14.452	90,080	
Proportion of test results <2 mg/L	70%	15%	
Median CRP	1	7	<0.001
25th percentile	0.8	3.4	
75th percentile	2.5	12	
Mean CRP	2.75	8.08	
CRP (SD)	3.78	5.56	
Comorbidities at baseline			
Hypertension (*n*, %)	3.068 (50%)	7.224 (55%)	<0.001
Atrial fibrillation (*n*, %)	1.058 (17%)	3.288 (25%)	<0.001
Stroke (*n*, %)	1.483 (24%)	3.160 (24%)	>0.9
AMI (*n*, %)	377 (6%)	741 (6%)	0.2
COPD (*n*, %)	808 (13%)	2.95 (23%)	<0.001
Heart failure (*n*, %)	1.162 (19%)	3.840 (29%)	<0.001
Type 2 diabetes (*n*, %)	1.143 (19%)	2.870 (22%)	<0.001

SI, systemic inflammation; ASCVD, atherosclerotic cardiovascular disease; CKD, chronic kidney disease; CRP, C-reactive protein; AMI, acute myocardial infarction; COPD, chronic obstructive pulmonary disease.

### Burden of illness associated with SI

**Figure 2:**
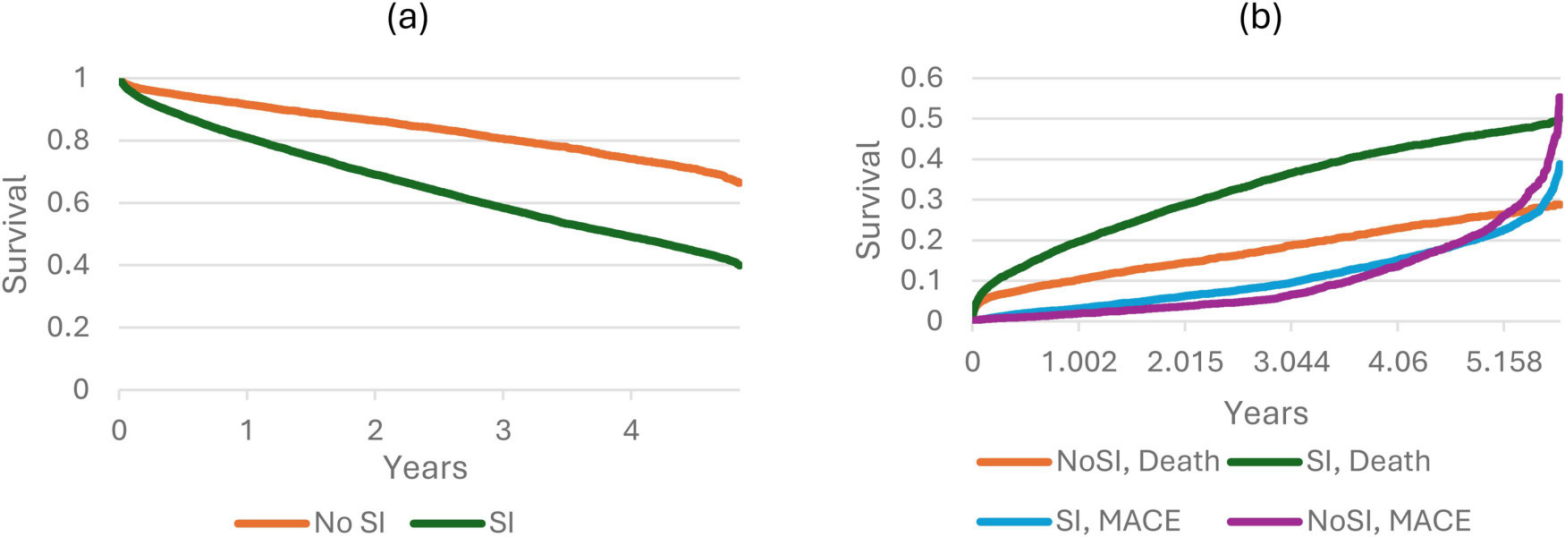
Kaplan–Meier plot for all-cause mortality **(a)**, and cumulative probability plot for MACE and all-cause mortality **(b)**.

Table 3 presents the hazard from the Cox regressions. The table presents the results from an unadjusted model as well as the final model for the overall effect and by subgroups. The table contains the HR for mortality as the main outcome, and the HR for ‘MACE or death’. Hazard ratios from stepwise models are presented in Supplementary Table S2. Kaplan–Meier plots and cumulative probability distributions for MACE and all-cause mortality are presented in Figures 2a,b. The cumulative probability distributions are based on a competing risk approach, allowing separate visualisation and interpretation of MACE and mortality.

**Table 3:** Survival and MACE, total and subgroup analyses.

Exposure	Univariate		Final	
	HR	95% CI	HR	95% CI
Survival				
HR SI overall	2.35	2.22–2.50	2.06	1.92–2.21
Sex				
HR SI men			2.46	2.22–2.71
HR SI women			1.72	1.56–1.89
Comorbidities				
HR SI heart failure			2.08	1.84–2.35
HR SI no heart failure			2.05	1.89–2.23
MACE				
HR SI overall	1.9	1.81–2.01	1.66	1.57–1.77
Sex				
HR SI men			1.62	1.5–1.75
HR SI women			1.32	1.22–1.43
Comorbidities				
HR SI heart failure			1.63	1.47–1.82
HR SI no heart failure			1.41	1.32–1.51
	SI	No SI	SI	No SI
*N*	12.524	5.932	9.742	3.835
Events (mortality)	7.224	2.217	5.857	1.634
Median observation time (years)	2.76	5.7	2.69	5.25

Final model adjusted for sex, age, education, region of residence, history of heart failure, HbA1c, LDL-c and triglyceride.

In the final multivariate-adjusted model, SI was associated with a 106% higher risk of death (HR: 2.06, CI: 1.92–2.21) and the hazard of ‘MACE or death’ by 66% (HR: 1.66, CI: 1.57–1.77). SI was associated with a higher risk of death and MACE in all analyses and subgroups; however, a significant difference in the risk of SI associated with mortality and MACE was estimated within the subgroups.

**Figure 3:**
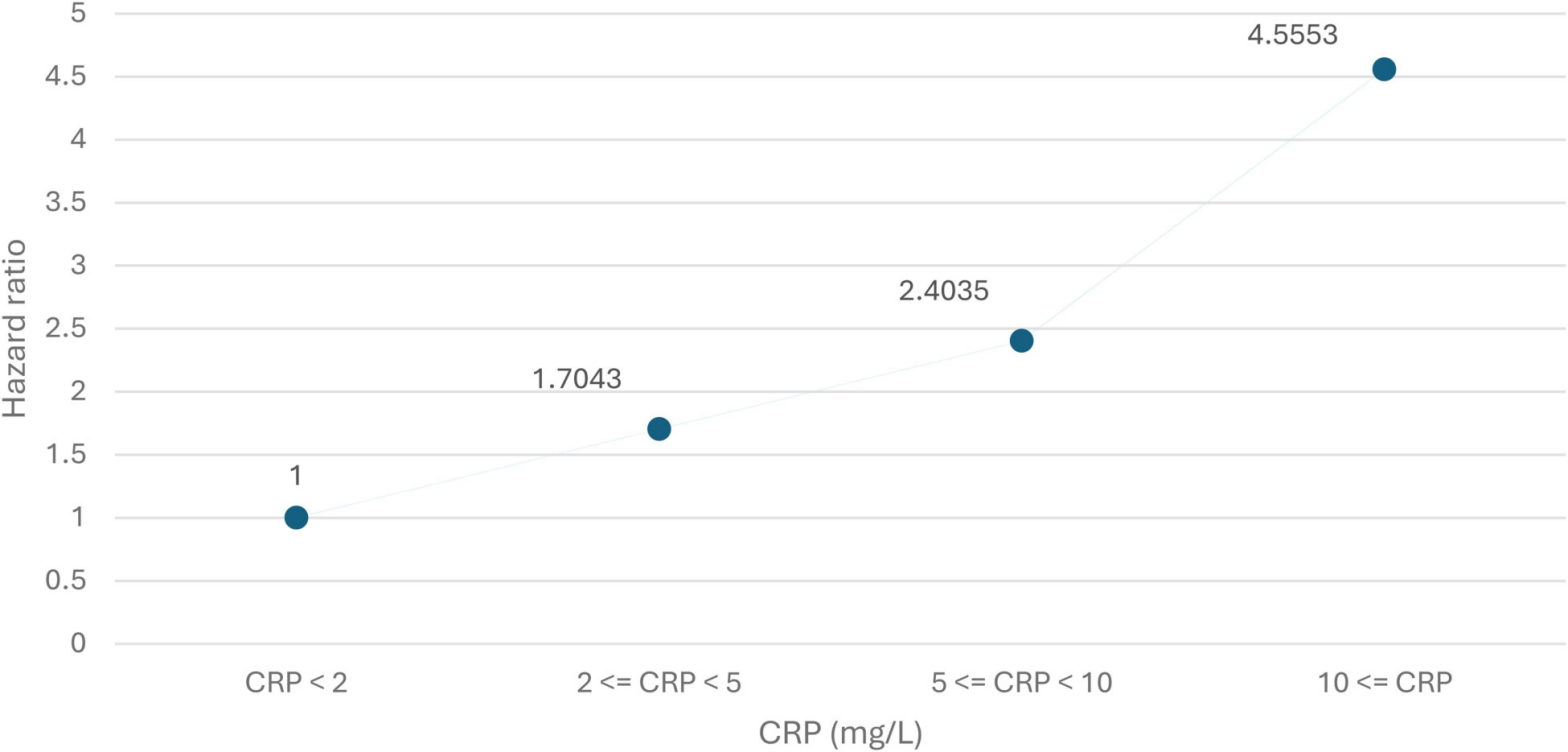
Hazard ratios from repeated measurement of Cox regression with clustered CRP-test results, including confidence intervals. Hazard ratios estimated via repeated measurement of Cox proportional hazard regression, with CRP <2 mg/L as a reference.

The results from the repeated measurement of Cox regression to account for potentially changing inflammation status during the study are presented in Figure 3. There is a clear gradient demonstrating increasing hazard of all-cause mortality as CRP levels increase. Compared to CRP <2 mg/dL, CRP of ≥2 mg/L to <5 mg/L, CRP of of ≥5 mg/L to <10 mg/L and CRP ≥10 mg/L increased the hazard of death by 70% (HR: 1.7, CI: 1.52–1.90), 140% (HR: 2.4, CI: 2.17–2.66) and 356% (HR: 4.56, CI: 4.14–5.02), respectively.

In Supplementary Tables S3, S4 the results using alternative definitions of SI is presented. In Supplementary Table S1, SI is defined by having one CRP test result of 2 mg/L–20 mg/L within two years of index date. In Supplementary Table S2, the analysis was based on an SI definition where three CRP test result of 2 mg/L–20 mg/L less than six months apart was observed within two years of index date. The results from these analyses does not change the inference we make from the results of the main analysis.

A total of 104,532 valid CRP test results were observed during the study period. Individuals with SI were tested about three times more frequently than were those with no SI. To investigate whether this difference in test frequency influenced the results, a model was developed that included the variable ‘number of tests.’ The effect was statistically significant, but with a hazard ratio (HR) of 1.03 [CI: 1.03–1.03] which is low compared to the found hazard ratios in the study.

## Discussion

This large nation-wide study investigates the risk of death associated with SI among patients with concomitant ASCVD and CKD. The analysis found that individuals diagnosed with both ASCVD and CKD stage 3–4 had a 2.4-fold increased hazard of all-cause mortality in the presence of SI compared to those without SI. Individuals with SI experience more comorbidities than those with no SI at index date, but after adjusting for person characteristics, the hazard of all-cause mortality was still 2.1-fold higher for individuals with SI. Results were consistent in subgroup analyses. Moreover, there was a consistent gradient in the association between CRP level and hazard of death.

The findings align with the existing literature. Burger et al. (13) found that a CRP increase of 1 mg/L increased the hazard of reoccurring CVD event by 8% (HR: 1.08), while Cho et al. (12) found that one standard deviation difference in CRP resulted in an 11% increase in risk of recurring coronary artery disease. In Denmark, Doi et al. (14) found a CRP hazard ratio of 1.3 for all-cause mortality; however, that result reflects the general population participating in a screening program rather than a population with both ASCVD and CKD as in this study. When a population at high risk for atherosclerotic disease was considered in Ridker et al. (15) an all-cause mortality hazard ratio related to CRP of 2.42 was found. While CRP levels can fluctuate over short timeframes, limiting their reliability for individual-level decision-making, it remains a well-established biomarker of inflammation in population-level studies. To mitigate the potential impact of short-term variability, the SI classification used in this study was based on multiple CRP tests taken over time.

In the current study, CRP was observed when individuals sought healthcare services. This introduced a bias, where a CRP test may show indication of SI when in fact the elevated CRP is a natural response to a particular incident rather than a true SI diagnosis. To mitigate this bias and improve the robustness of the results, we require individuals in the SI group to have at least two tests recorded within six months. No minimum time between CRP tests was implemented, but given the short half-life of CRP, transient elevations due to acute conditions are unlikely to persist across multiple measurements.

In addition, most of the previous studies used only one CRP test result to classify SI. As demonstrated in the sensitivity analysis (Supplementary Tables S3, S4), the definition of SI influences the results. When only one CRP test resulting in the 2 mg/L–20 mg/L range is required, the hazard ratio for all-cause mortality is lower than in the main analysis. When three tests within the same range are required, the hazard ratio increases. This could indicate some misclassification in the SI/no-SI study groups. Despite this potential misclassification, the inference from having SI does not change from the main analysis. This is particularly evident in the repeated measurement analysis, where assumptions regarding SI are omitted. In that analysis, we observed a clear gradient demonstrating the association between elevated CRP and mortality (Figure 3). Future studies could build on this gradient by examining potential non-linear relationships between elevated CRP levels and mortality using spline-based methods.

A limitation of the study is the absence of a precise onset date for SI. To account for the systemic nature of the inflammation, we required two CRP tests indicative of SI within two years of the latter diagnosis of ASCVD and CKD. Relying on CRP tests taken during the follow-up period may imply that for some individuals, the follow-up could begin prior to the first test indicative of SI. However, given that SI is a condition that can persist or recur, pinpointing the exact onset is challenging, which is why we anchored our analyses in the latter diagnosis of ASCVD and CKD. Additionally, it is plausible that individuals may have experienced SI for some time before undergoing CRP tests, as this can be influenced by personal health-seeking behaviours, which could potentially lead to individuals with SI being excluded from the study population.

Another implication of using CRP tests conducted during follow-up to identify systemic inflammation is that immortal time bias may affect the results, as only patients who survived from the index date to the time of CRP testing would be included. However, this bias is likely to lead to an underestimation of hazard ratios, as individuals who potentially had SI but died before the tests were performed are excluded from the SI group. It would therefore not affect the conclusions of the study, which indicate that SI is associated with a higher risk of mortality.

A weakness of the study is the lack of information around cause of death. The cumulative probability of MACE did not differ in a noteworthy way between the two groups. This is likely due to some MACE incidences resulting in death, which might not be recorded with an ICD-10 code in the registers prior to the date of death. A topic for future research would therefore be to investigate 1) whether MACE occurs with the same frequency in the two populations, and 2) whether SI is associated with severity of MACE, should MACE occur.

As this is a register study, it suffers from the common issue of not being able to validate all included observations. However, the Danish national registers are frequently applied in research and are generally considered to be accurate and of high quality. Furthermore, there is the possibility of misclassification of SI status. Individuals who are sicker will have a higher frequency of testing, which in turn will increase the probability of being classified as having SI. We did observe this effect in the sensitivity analysis. However, the effect estimate is small with a narrow confidence interval (HR: 1.03 CI: 1.03–1.03). Furthermore, the main results are consistent with the results we observe when allowing for CRP levels to change over time (Figure 3). This should therefore not influence the inference we can draw from the results.

Taken together, these findings suggest that systemic inflammation captures an additional dimension of cardiovascular risk beyond established factors in patients with ASCVD and CKD. While CRP is not intended for individual-level decision-making in routine care, future work could focus on developing prognostic models that incorporate inflammatory markers alongside conventional risk factors.

## Conclusion

Among patients with both ASCVD and CKD, systemic inflammation, defined by a CRP level of ≥2 mg/L, is highly prevalent and associated with more than a twofold increase in the risk of mortality. These findings underscore the potential of systemic inflammation as a critical marker for identifying individuals with ASCVD and CKD at exceptionally high risk, who may benefit from targeted preventive interventions.

## Data Availability

The datasets presented in this article are not readily available because this study utilizes Danish administrative register data, which are governed by strict privacy regulations. As such, the data cannot be made available for public access. Requests to access the datasets should be directed to Kasper Lundgren, kasper.loewe.lundgren@parthenon.ey.com.

## Supplementary material

Supplementary Table S1 Exclusion criteria—conditions thought to influence CRP levels. Includes all sublevels of the specified ICD-10 codes.

Supplementary Table S2 Summary table of step-models—HR for mortality with 2 CRP tests for SI.

Supplementary Table S3 HR for mortality or mortality and MACE with alternative criteria for identifying SI.

Supplementary Table S4 Definition of selected comorbidities. Includes all sublevels of the specified ICD-10 codes.

## References

[1] Vos T Lim SS Abbafati C Abbas KM Abbasi M Abbasifard M Global burden of 369 diseases and injuries in 204 countries and territories, 1990–2019: a systematic analysis for the global burden of disease study 2019. Lancet. (2020) 396(10258):1204–22. 10.1016/S0140-6736(20)30925-9 33069326 PMC7567026

[2] Bikbov B Purcell CA Levey AS Smith M Abdoli A Abebe M Global, regional, and national burden of chronic kidney disease, 1990–2017: a systematic analysis for the global burden of disease study 2017. Lancet Lond Engl. (2020) 395(10225):709–33. 10.1016/S0140-6736(20)30045-3 PMC7049905 32061315

[3] Mathew RO Bangalore S Lavelle MP Pellikka PA Sidhu MS Boden WE Diagnosis and management of atherosclerotic cardiovascular disease in chronic kidney disease: a review. Kidney Int. (2017) 91(4):797–807. 10.1016/j.kint.2016.09.049 28040264

[4] Christensen MB Jimenez-Solem E Ernst MT Schmidt M Pottegård A Grove EL. Low-dose aspirin for primary and secondary prevention of cardiovascular events in Denmark 1998–2018. Sci Rep. (2021) 11(1):13603. 10.1038/s41598-021-93179-8 34193948 PMC8245534

[5] Lawler PR Bhatt DL Godoy LC Lüscher TF Bonow RO Verma S Targeting cardiovascular inflammation: next steps in clinical translation. Eur Heart J. (2021) 42(1):113–31. 10.1093/eurheartj/ehaa099 32176778

[6] Crea F Libby P. Acute coronary syndromes: the way forward from mechanisms to precision treatment. Circulation. (2017) 136(12):1155–66. 10.1161/CIRCULATIONAHA.117.029870 28923905 PMC5679086

[7] Lindh M Banefelt J Fox KM Hallberg S Tai MH Eriksson M Cardiovascular event rates in a high atherosclerotic cardiovascular disease risk population: estimates from Swedish population-based register data. Eur Heart J Qual Care Clin Outcomes. (2019) 5(3):225–32. 10.1093/ehjqcco/qcy058 30649251 PMC6613595

[8] Chen S Hsu WY Lin YN Wang CY Wu CH Chang KH. Incidence and risk of major adverse cardiovascular events in middle-aged patients with chronic kidney disease: a population-based cohort study. Int Urol Nephrol. (2019) 51(7):1219–27. 10.1007/s11255-019-02157-7 31020627

[9] Wong ND Budoff MJ Ferdinand K Graham IM Michos ED Reddy T Atherosclerotic cardiovascular disease risk assessment: an American society for preventive cardiology clinical practice statement. Am J Prev Cardiol. (2022) 10:100335. 10.1016/j.ajpc.2022.100335 35342890 PMC8943256

[10] Alfaddagh A Martin SS Leucker TM Michos ED Blaha MJ Lowenstein CJ Inflammation and cardiovascular disease: from mechanisms to therapeutics. Am J Prev Cardiol. (2020) 4:100130. 10.1016/j.ajpc.2020.100130 34327481 PMC8315628

[11] Mainous AG Sharma P Jo A. Systemic inflammation among adults with diagnosed and undiagnosed cardiometabolic conditions: a potential missed opportunity for cardiovascular disease prevention. Front Med (Lausanne). (2023) 10:1327205. 10.3389/fmed.2023.1327205 38274464 PMC10808594

[12] Cho SMJ Koyama S Honigberg MC Surakka I Haidermota S Ganesh S Genetic, sociodemographic, lifestyle, and clinical risk factors of recurrent coronary artery disease events: a population-based cohort study. Eur Heart J. (2023) 44:ehad380. 10.1093/eurheartj/ehad380 PMC10516626 37350734

[13] Burger PM Pradhan AD Dorresteijn JAN Koudstaal S Teraa M De Borst GJ C-reactive protein and risk of cardiovascular events and mortality in patients with various cardiovascular disease locations. Am J Cardiol. (2023) 197:13–23. 10.1016/j.amjcard.2023.03.025 37218417

[14] Doi T Langsted A Nordestgaard BG. Dual elevated remnant cholesterol and C-reactive protein in myocardial infarction, atherosclerotic cardiovascular disease, and mortality. Atherosclerosis. (2023) 379:117141. 10.1016/j.atherosclerosis.2023.05.010 37217436

[15] Ridker PM Bhatt DL Pradhan AD Glynn RJ MacFadyen JG Nissen SE. Inflammation and cholesterol as predictors of cardiovascular events among patients receiving statin therapy: a collaborative analysis of three randomised trials. Lancet. (2023) 401(10384):1293–301. 10.1016/S0140-6736(23)00215-5 36893777

[16] Vrints C Andreotti F Koskinas KC Rossello X Adamo M Ainslie J 2024 ESC guidelines for the management of chronic coronary syndromes. Eur Heart J. (2024) 45:ehae177. 10.1093/eurheartj/ehae177 39210710

[17] Ridker PM Tuttle KR Perkovic V Libby P MacFadyen JG. Inflammation drives residual risk in chronic kidney disease: a CANTOS substudy. Eur Heart J. (2022) 43(46):4832–44. 10.1093/eurheartj/ehac444 35943897

[18] Mazhar F Faucon AL Fu EL Szummer KE Mathisen J Gerward S Systemic inflammation and health outcomes in patients receiving treatment for atherosclerotic cardiovascular disease. Eur Heart J. (2024) 45(44):4719–30. 10.1093/eurheartj/ehae557 39211962 PMC11578643

[19] Overview of Mandatory Reporting. Danish Research Ethics Committees—part of the Danish National Center for Ethics. Copenhagen: Danish Research Ethics Committees (2024). Available online at: https://researchethics.dk/information-for-researchers/overview-of-mandatory-reporting (Accessed May 20, 2025)

[20] Han E Fritzer-Szekeres M Szekeres T Gehrig T Gyöngyösi M Bergler-Klein J. Comparison of high-sensitivity C-reactive protein vs C-reactive protein for cardiovascular risk prediction in chronic cardiac disease. J Appl Lab Med. (2022) 7(6):1259–71. 10.1093/jalm/jfac069 36136302

[21] Kildemoes HW Sørensen HT Hallas J. The Danish national prescription registry. Scand J Public Health. (2011) 39(7_Suppl):38–41. 10.1177/1403494810394717 21775349

[22] Lynge E Sandegaard JL Rebolj M. The Danish national patient register. Scand J Public Health. (2011) 39(7_Suppl):30–3. 10.1177/1403494811401482 21775347

[23] Arendt JFH Hansen AT Ladefoged SA Sørensen HT Pedersen L Adelborg K. Existing data sources in clinical epidemiology: laboratory information system databases in Denmark. Clin Epidemiol. (2020) 12:469–75. 10.2147/CLEP.S245060 32547238 PMC7244445

[24] Ridker PM Everett BM Thuren T MacFadyen JG Chang WH Ballantyne C Antiinflammatory therapy with canakinumab for atherosclerotic disease. N Engl J Med. (2017) 377(12):1119–31. 10.1056/NEJMoa1707914 28845751

[25] Ridker PM Danielson E Fonseca FAH Genest J Gotto AM Kastelein JJP Rosuvastatin to prevent vascular events in men and women with elevated C-reactive protein. N Engl J Med. (2008) 359(21):2195–207. 10.1056/NEJMoa0807646 18997196

[26] Helweg-Larsen K. The Danish register of causes of death. Scand J Public Health. (2011) 39(7_Suppl):26–9. 10.1177/1403494811399958 21775346

[27] Falkentoft AC Gerds TA Zareini B Knop FK Køber L Torp-Pedersen C Risk of first-time major cardiovascular event among individuals with newly diagnosed type 2 diabetes: data from Danish registers. Diabetologia. (2023) 66(11):2017–29. 10.1007/s00125-023-05977-6 37528178 PMC10541344

[28] Attar R Valentin JB Andell P Nielsen RE Jensen SE. Major adverse cardiovascular events following acute coronary syndrome in patients with bipolar disorder. Int J Cardiol. (2022) 363:1–5. 26. 10.1016/j.ijcard.2022.06.036 35716946

[29] Ai C Norton EC. Interaction terms in logit and probit models. Econ Lett. (2003) 80(1):123–9. 10.1016/S0165-1765(03)00032-6

