# Supplementary material for: Systemic inflammation is associated with increased risk of death in population with atherosclerotic cardiovascular disease and chronic kidney disease—a Danish national register study
Source: Front Cardiovasc Med. 2026 Feb 27;13:1749835. doi: 10.3389/fcvm.2026.1749835 (PMC12982448; doi:10.3389/fcvm.2026.1749835)
Supplement: Supplementary Table S1 — Exclusion criteria—conditions thought to influence CRP levels. Includes all sublevels of the specified ICD-10 codes. [file Table1.docx]

**Table S1:** Exclusion criteria – conditions thought to influence CRP levels

| **Condition** | **Identification** |  |
| --- | --- | --- |
| HIV | ICD-10: B20-B24, Z21 |  |
| Hepatitis C | ICD-10: B11, B18.2 |  |
| Tuberculosis | ICD-10: A15–A19 |  |
| Inflammatory bowel disease | ICD-10: K58 |  |
| Cancer | ICD-10: C chapter and D00–D48, except C61.9 (prostate cancer), C43–C44 (skin cancer) and D06 (carcinoma of the cervix) |  |
| Transplant | ICD-10: N16.5, T81.7E1, T82.3, T82.7B, T83.2, T83.5C, T83.6C, T86.0, T86.1–T86.9, Z94 | |

Note: Includes all sublevels of the specified ICD-10 codes.
