# Supplementary material for: Systemic inflammation is associated with increased risk of death in population with atherosclerotic cardiovascular disease and chronic kidney disease—a Danish national register study
Source: Front Cardiovasc Med. 2026 Feb 27;13:1749835. doi: 10.3389/fcvm.2026.1749835 (PMC12982448; doi:10.3389/fcvm.2026.1749835)
Supplement: Supplementary Table S2 — Summary table of step-models—HR for mortality with 2 CRP tests for SI. [file Table2.docx]

**Table S2:** Summary table of step-models – HR for mortality with 2 CRP tests for SI

| Exposure | Demographics | | Comorbidities | | HbA1c | | LDL-c | | Triglyceride | |
| --- | --- | --- | --- | --- | --- | --- | --- | --- | --- | --- |
|  | HR | 95% CI | HR | 95% CI | HR | 95% CI | HR | 95% CI | HR | 95% CI |
| **Survival** |  |  |  |  |  |  |  |  |  |  |
| HR SI overall | 2.24 | 2.12 - 2.38 | 2.14 | 2.02 - 2.27 | 2.03 | 1.90 - 2.17 | 2.12 | 1.99 - 2.27 | 2.14 | 2.00 - 2.28 |
| Sex |  |  |  |  |  |  |  |  |  |  |
| HR SI men | 2.66 | 2.44 - 2.9 | 2.53 | 2.32 - 2.76 | 2.4 | 2.19 - 2.64 | 2.47 | 2.25 - 2.72 | 2.5 | 2.28 - 2.74 |
| HR SI women | 1.9 | 1.75 - 2.06 | 1.82 | 1.68 - 1.97 | 1.71 | 1.57 - 1.87 | 1.81 | 1.65 - 1.98 | 1.82 | 1.66 - 1.99 |
| Comorbidities |  |  |  |  |  |  |  |  |  |  |
| HR SI heart failure |  |  | 2.08 | 1.86 - 2.32 | 2.06 | 1.83 - 2.32 | 2.12 | 1.89 - 2.39 | 2.17 | 1.93 - 2.44 |
| HR SI no heart failure |  |  | 2.17 | 2.02 - 2.33 | 2.02 | 1.87 - 2.18 | 2.12 | 1.96 - 2.29 | 2.12 | 1.97 - 2.29 |
| HR SI type 2 diabetes |  |  | 2.36 | 2.08 - 2.68 |  |  | 2.29 | 2 - 2.62 | 2.3 | 2.02 - 2.62 |
| HR SI no type 2 diabetes |  |  | 2.08 | 1.95 - 2.23 |  |  | 2.07 | 1.92 - 2.23 | 2.09 | 1.94 - 2.25 |
| **MACE** |  |  |  |  |  |  |  |  |  |  |
| HR SI overall | 1.83 | 1.74 - 1.93 | 1.77 | 1.68 - 1.87 | 1.66 | 1.57 - 1.76 | 1.72 | 1.62 - 1.82 | 1.73 | 1.64 - 1.83 |
| Sex |  |  |  |  |  |  |  |  |  |  |
| HR SI men | 1.78 | 1.66 - 1.9 | 1.72 | 1.61 - 1.84 | 1.64 | 1.52 - 1.76 | 1.64 | 1.53 - 1.76 | 1.67 | 1.56 - 1.8 |
| HR SI women | 1.49 | 1.39 - 1.59 | 1.45 | 1.36 - 1.55 | 1.33 | 1.23 - 1.43 | 1.41 | 1.31 - 1.53 | 1.42 | 1.32 - 1.53 |
| Comorbidities |  |  |  |  |  |  |  |  |  |  |
| HR SI heart failure |  |  | 1.7 | 1.55 - 1.87 | 1.64 | 1.48 - 1.82 | 1.67 | 1.51 - 1.85 | 1.71 | 1.55 - 1.89 |
| HR SI no heart failure |  |  | 1.55 | 1.46 - 1.64 | 1.43 | 1.34 - 1.52 | 1.48 | 1.39 - 1.57 | 1.49 | 1.41 -1.59 |
| HR SI type 2 diabetes |  |  | 1.69 | 1.53 - 1.88 |  |  | 1.61 | 1.44 - 1.8 | 1.63 | 1.46 - 1.82 |
| HR SI no type 2 diabetes |  |  | 1.56 | 1.48 - 1.65 |  |  | 1.51 | 1.42 - 1.6 | 1.53 | 1.44 - 1.62 |
|  | SI | No SI | SI | No SI | SI | No SI | SI | No SI | SI | No SI |
| N | 12,524 | 5,932 | 12,524 | 5,932 | 10,972 | 4,323 | 10,906 | 4,660 | 11,813 | 5,110 |
| Events (mortality) | 7,224 | 2,217 | 7,224 | 2,217 | 6,511 | 1,795 | 6,297 | 1,824 | 6650 | 1,924 |
| Median observation time (years) | 2.76 | 5.7 | 2.76 | 5.7 | 2.59 | 5.15 | 2.93 | 5.66 | 2.92 | 5.66 |
