# Supplementary material for: Systemic inflammation is associated with increased risk of death in population with atherosclerotic cardiovascular disease and chronic kidney disease—a Danish national register study
Source: Front Cardiovasc Med. 2026 Feb 27;13:1749835. doi: 10.3389/fcvm.2026.1749835 (PMC12982448; doi:10.3389/fcvm.2026.1749835)
Supplement: Supplementary Table S3 — HR for mortality or mortality and MACE with alternative criteria for identifying SI. [file Table3.docx]

**Table S3:** HR for mortality or mortality and MACE with alternative criteria for identifying SI

|  | One CRP-test to determine SI | | | | Three CRP-tests to determine SI | | | |
| --- | --- | --- | --- | --- | --- | --- | --- | --- |
| Exposure | Univariate | | Final | | Univariate | | Final | |
|  | HR | 95% CI | HR | 95% CI | HR | 95% CI | HR | 95% CI |
| **Survival** |  |  |  |  |  |  |  |  |
| HR SI overall | 2.65 | 2.40 – 2.92 | 2.25 | 1.99 – 2.55 | 2.08 | 1.99 – 2.18 | 1.87 | 1.78 – 1.98 |
| **Sex** |  |  |  |  |  |  |  |  |
| HR SI men |  |  | 2.56 | 2.14 – 3.07 |  |  | 2.11 | 1.96 – 2.27 |
| HR SI women |  |  | 1.97 | 1.66 – 2.35 |  |  | 1.65 | 1.53 – 1.78 |
| **Comorbidities** |  |  |  |  |  |  |  |  |
| HR SI heart failure |  |  | 2.35 | 1.87 – 2.95 |  |  | 1.78 | 1.63 – 1.95 |
| HR SI no heart failure |  |  | 2.21 | 1.90 – 2.57 |  |  | 1.93 | 1.80 – 2.06 |
| **MACE** |  |  |  |  |  |  |  |  |
| HR SI overall | 2.04 | 1.88 – 2.22 | 1.65 | 1.49 – 1.83 | 1.80 | 1.72 – 1.88 | 1.64 | 1.56 – 1.72 |
| **Sex** |  |  |  |  |  |  |  |  |
| HR SI men |  |  | 1.54 | 1.36 – 1.75 |  |  | 1.6 | 1.50 – 1.71 |
| HR SI women |  |  | 1.31 | 1.15 – 1.49 |  |  | 1.37 | 1.28 – 1.46 |
| **Comorbidities** |  |  |  |  |  |  |  |  |
| HR SI heart failure |  |  | 1.82 | 1.50 – 2.21 |  |  | 1.52 | 1.40 – 1.65 |
| HR SI no heart failure |  |  | 1.32 | 1.19 – 1.46 |  |  | 1.47 | 1.39 – 1.55 |
