# Supplementary material for: Systemic inflammation is associated with increased risk of death in population with atherosclerotic cardiovascular disease and chronic kidney disease—a Danish national register study
Source: Front Cardiovasc Med. 2026 Feb 27;13:1749835. doi: 10.3389/fcvm.2026.1749835 (PMC12982448; doi:10.3389/fcvm.2026.1749835)
Supplement: Supplementary Table S4 — Definition of selected comorbidities. Includes all sublevels of the specified ICD-10 codes. [file Table4.docx]

**Table S4:** Definition of selected comorbidities

| **Condition** | **Identification** |
| --- | --- |
| Stroke | ICD-10: I63.9 |
| Heart failure | ICD-10: I50.9 |
| Type 2 diabetes | Identified as ”TYPE 2” in Register of Selected Chronic Diseases |
| Type 1 diabetes | Identified as ”TYPE 1” in Register of Selected Chronic Diseases |
| Chronic obstructive pulmonary disease | ICD-10: J44.0, J44.1, J44.9, J45.9 |
| Myocardial infarction | ICD-10: I21.9 |
| Atrial fibrillation | ICD-10: I48.9 |

Note: Includes all sublevels of the specified ICD-10 codes.
